# Incretin-FGF21 fusion molecule maximizes metabolic effects in mice: Incretin-FGF21 maximizes metabolic effects in mice

**DOI:** 10.3724/abbs.2023276

**Published:** 2023-12-14

**Authors:** Yulin Kong, Shenglong Zhu, Yong Q. Chen

**Affiliations:** 1 Department of Urology Jiangnan University Medical Center Wuxi 214122 China; 2 Wuxi School of Medicine Jiangnan University Wuxi 214122 China

Common metabolic diseases, such as obesity and nonalcoholic steatohepatitis (NASH), are major public health concerns. The only therapeutic approach that offers weight loss of 20% or more is bariatric surgery. Medications are now available, such as liraglutide (Victoza), dulaglutide (Trulicity) and semaglutide (Ozempic) which are gut hormone incretin memetics [1]. Noticeably, the glucose-dependent insulinotropic polypeptide receptor (GIPR)/glucagon-like peptide 1 receptor (GLP-1R) dual agonist tirzepatide (Mounjaro) attains a greater than 20% reduction in body weight [2]. Therefore, incretin receptor agonists are being extensively explored [1].

Hepatic hormone fibroblast growth factor 21 (FGF21) is a key metabolic regulator [3]. The FGF21 analog is promising for the treatment of NASH [4]. In contrast to incretin-incretin peptides which are dual agonists of the incretin receptor family, we explored the metabolic benefits of incretin-FGF21 fusion proteins which target both incretin and FGF21 receptor families. We expressed pro21, an IgG _1_ Fc-FGF21 fusion protein which is similar to AKR-001 (Akero Therapeutics, San Francisco, USA) in *Escherichia coli*, and expressed euk21 (an IgG _4_ Fc-modified human FGF21 fusion protein; DDKJ Biomedicals, Wuxi, China), L21 (a liraglutide-IgG _4_ Fc-modified human FGF21 fusion protein), LG21 (a liraglutide-GIP-IgG _4_ Fc-modified human FGF21 fusion protein), D21 (a dulaglutide-IgG _4_ Fc-modified human FGF21 fusion protein), DG21 (a dulaglutide-GIP-IgG _4_ Fc-modified human FGF21 fusion protein), and G21 (a GIP-IgG _4_ Fc-modified human FGF21 fusion protein) in mammalian cells ( Supplementary Figure S1). We treated diet-induced obese (DIO) mice with incretin-FGF21 fusion proteins (25 nmol/kg, once a week, subcutaneous injection) for 7 weeks and then monitored glycemic and lipid parameters, body weight, and liver function. Pro21 and euk21 were used as controls for FGF21, and dulaglutide (Trulicity; Eli Lilly, Indianapolis, USA) was used as a control for GLP-1.

The first dose administration of Trulicity and triple agonist (LG21 and DG21) resulted in significant decreases in blood glucose levels ( Figure 1A). Mono (Trulicity, pro21, and euk21) and dual (L21 and D21) agonists significantly reduced fasting blood glucose levels ( Figure 1B). Mono (euk21) and dual (L21 and D21) agonist treatment also significantly increased intraperitoneal glucose tolerance ( Figure 1C) and insulin sensitivity ( Figure 1D). However, the effect of triple agonists (LG21 and DG21) largely disappeared after multiple-dose administration ( Figure 1). These data suggested that dulaglutide alone or in combination with FGF21 can effectively improve glucose metabolism. Although D21 seems to be less effective than Trulicity ( Table 1; glycemic parameter score), the fact that Trulicity is a drug formulation and D21 is a pure protein may account for the slight difference.

**Figure 1:**
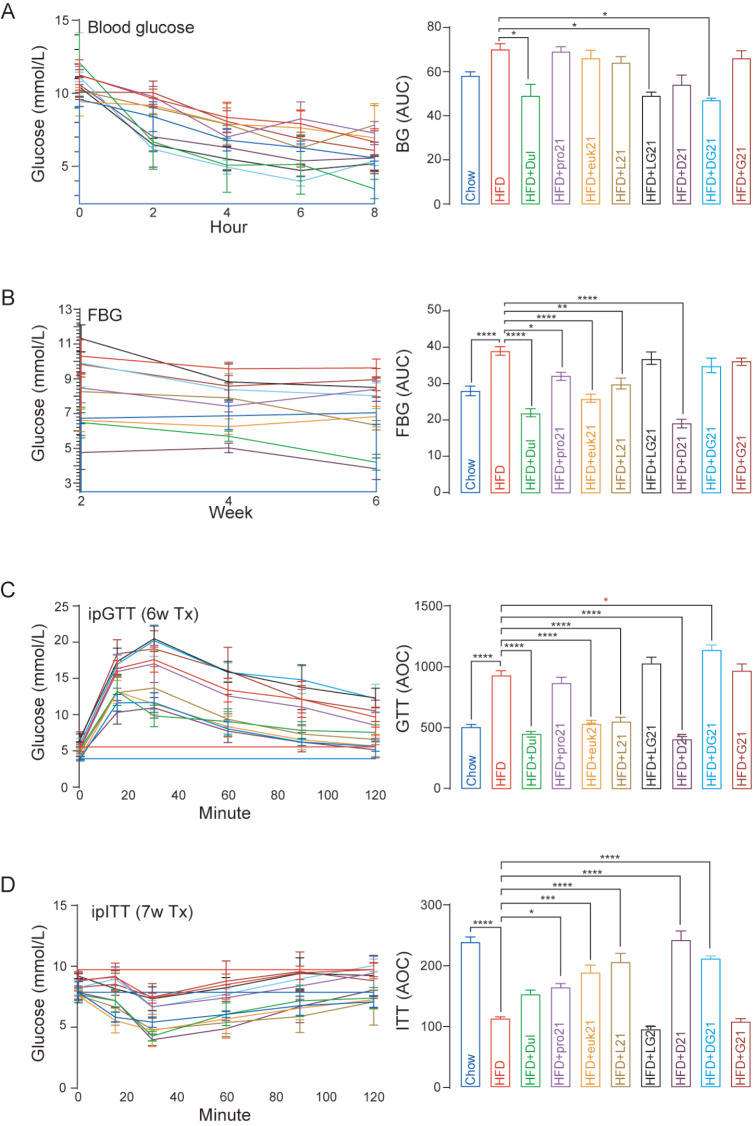
Figure 1 Effects of fusion proteins on glycemic metabolism (A) Blood glucose levels and area under the curve (AUC). (B) Fasting blood glucose levels and the area under the curve. (C) Intraperitoneal glucose tolerance test (ipGTT) and area of the curve (AOC). (D) Intraperitoneal insulin tolerance test (ipITT) and area of the curve. *P<0.05, **P<0.01, ***P<0.001, ****P<0.0001. BG: blood glucose, FBG: fasting blood glucose, Tx: treatment.

**Table:** **Table 1** Effects of incretin and FGF21 mimetics on mouse metabolism

Peptide	Improvement in glycemic parameters		Improvement in lipid parameters		Improvement in liver function parameters		Reduction in organ weight	Total score
BG	FBG	GTT	ITT	Score		B-TG	B-TC	B-LDL	L-TG	L-TC	L-LDL	Score		B-ALT	B-AST	A-ALP	L-ALT	L-AST	L-ALP	NAS	FibS	Score		Body	Liver	pgWAT	inWAT	Score
Trulicity	1.75	1.55	1.15	0	4.45		0.65	0.81	0.77	1.18	0	0.86	4.27		0.74	0	2.70	0.97	0.72	1.15	0.56	0.50	7.35		0.37	0.89	0	0	1.27	17.33
Prok21	0	0.64	0	0.40	1.04		0	0	0	0.45	0.74	0.38	1.57		0	0	0	0.15	0	0.91	0.75	0.42	2.23		0.26	0.43	0	0	0.69	5.53
euk21	0	1.18	0.94	0.60	2.73		0	0.64	0.72	1.38	0.94	0.73	4.41		0.99	0	2.74	1.02	1.11	1.32	0.78	0.58	8.54		0.67	0.98	0.55	0.46	2.66	18.33
L21	0	0.82	0.89	0.74	2.45		0	0	0.60	1.01	0.79	0.71	3.12		0.88	0	2.65	1.00	0.91	1.28	0.81	0.58	8.11		0.42	0.86	0	0	1.28	14.95
LG21	1.75	0	0	0	1.75		0	0	0	0	0	0.41	0.41		0	0	0	0	0	0	0.25	0.33	0.58		0	0	0	0	0.00	2.75
D21	0	1.82	1.25	1.03	4.09		0.79	0.96	0.88	1.18	0.79	0.93	5.54		1.05	0	3.55	1.01	0.89	1.07	1.00	0.58	9.15		0.68	0.99	0.76	0.59	3.02	21.80
DG21	1.92	0	–0.51	0.78	2.19		0	0	0	0	0	0	0		0	0	0	0	0	0	0.25	0.33	0.58		0	0	0	0	0.00	2.77
G21	0	0	0	0	0.00		0	–0.60	0	0	0	0	–0.60		0	0	0	0	0	0.60	0.25	0.42	1.26		0	0	0	0	0.00	0.66

No significant difference compared to the HFD group (score=0). Statistically different from HFD. Score=[Mean _HFD_-Mean _Tx_)/Mean _HFD_-Mean _chow_)]. BG: blood glucose, FBG: fasting blood glucose, GTT: glucose tolerance test, ITT: insulin tolerance test, B-TG: blood triacylglycerols, B-LDL: blood low density lipoprotein, L-TG: liver triacylglycerols, L-TC:liver total cholesterol, L-LDL: liver low density lipoprotein, B-ALT: blood alanine aminotransferase, B-AST: blood aspartate aminotransferase, B-ALP: blood alkaline pgosphatase, L-ALT:liver alanine aminotransferase; L-AST: liver aspartate aminotransferase, L-ALP: liver alkaline pgosphatase,NAS: nonalcogolic fatty disease activity score, FibS: fibrosis score, pgWAT: perigonadal white adipose tissue, inWAT: inguinal white adipose tissue.

Treatment with mono (Trulicity, pro21, and euk21) and dual (L21 and D21) agonists lowered the levels of triacylglycerol, total cholesterol, and low-density lipoprotein more prominently in the liver than in the blood ( Figure 2A). The dulaglutide-FGF21 (D21) fusion protein is the most effective, followed by euk21 and then Trulicity ( Table 1); hence, FGF21 appeared to have a greater impact than dulaglutide on lipid metabolism.

**Figure 2:**
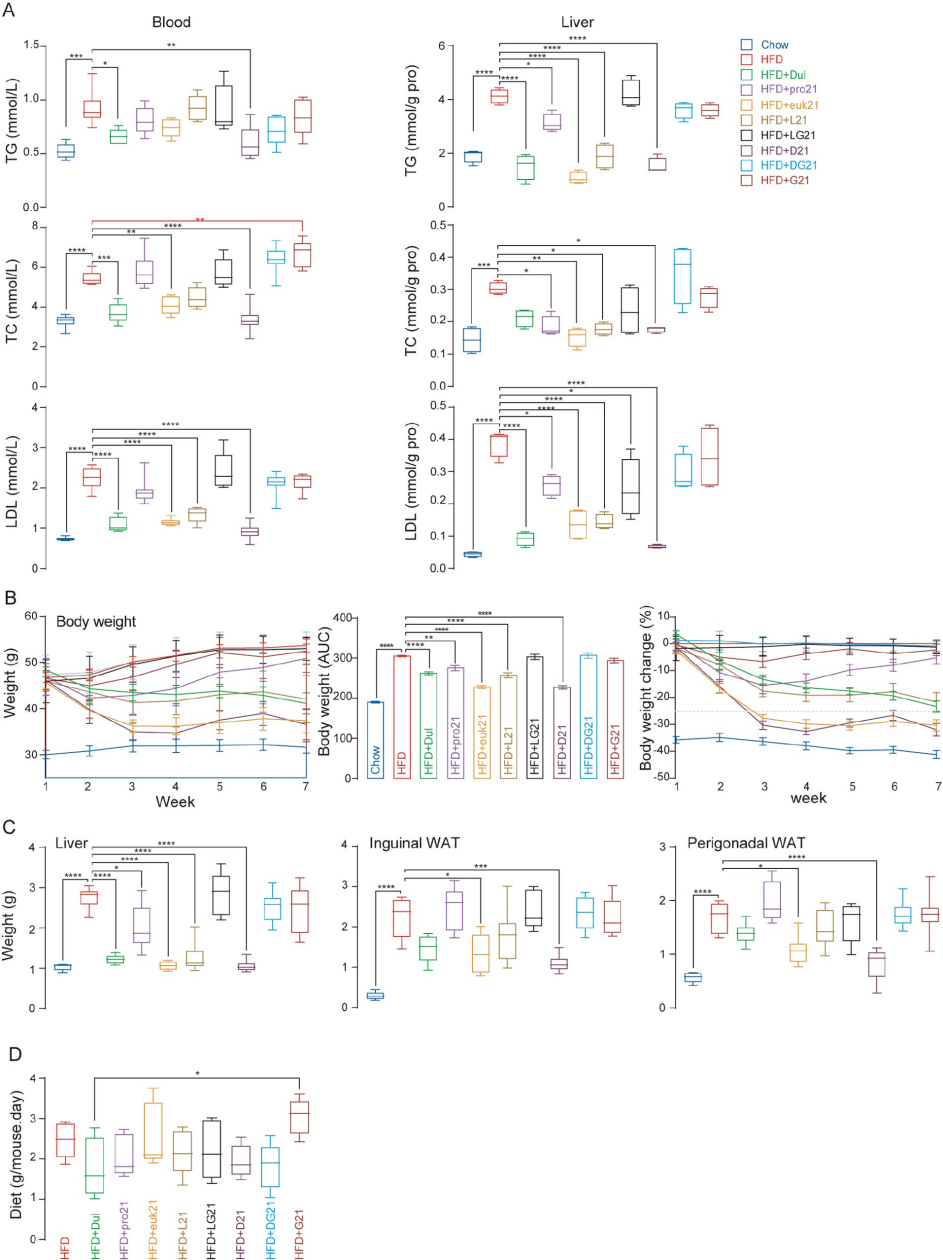
Figure 2 Effects of fusion proteins on lipid metabolism and organ weight (A) Box and whisker graph of blood and liver levels of triacylglycerols (TG), total cholesterol (TC), and low-density lipoprotein (LDL). (B) Body weight, area under the curve, and percent of body weight change. (C) Box and whisker graph of the liver, inguinal white adipose tissue (WAT) and perigonadal WAT weights. (D) Box and whisker graph of diet intake. Box and whisker graphs show the highest, the lowest, 25% and 75% quartile, and median values. *P<0.05, **P<0.01, ***P<0.001, ****P<0.0001.

Treatment with mono (Trulicity, pro21, and euk21) and dual (L21 and D21) agonists significantly reduced body and liver weights ( Figure 2B,C). However, reductions in adipose tissue weight were only significant in the groups with euk21 and D21 ( Figure 2C). Greater than 30% reductions in body weight were observed in the euk21- and D21-treated groups ( Figure 2B). FGF21 was seen to have a higher impact than dulaglutide on organ weights ( Table 1). No significant food intake difference was observed in the treated groups compared to the high-fat diet (HFD) group. However, a difference was noticeable between the Trulicity and G21 groups ( Figure 2D). Some studies have reported that GLP-1R [5] or GIPR [6] activation reduces food intake. It is unclear why G21 treatment leads to higher food intake than Trulicity treatment.

Mono (Trulicity and euk21) and dual (L21 and D21) agonist treatment significantly reduced liver levels of alanine aminotransferase (ALT), aspartate aminotransferase (AST) and alkaline phosphatase (ALP), as well as blood levels of ALT and ALP ( Supplementary Figure S2A) and nonalcoholic fatty liver score (NAS) and fibrosis score ( Supplementary Figure S2B). FGF21 again had a more marked effect than dulaglutide on liver function parameters ( Table 1).

In summary, we found that dulaglutide (Trulicity) had the most significant effect on reducing glycemic parameters ( Table 1). FGF21 alone (euk21) or in combination with dulaglutide (D21) had greater impacts than Trulicity on lipid metabolism, organ weights and liver functions. D21 had the most significant overall impact among the fusion proteins tested in our experiments ( Table 1). We noticed that eukaryotically expressed euk21 had a greater impact on metabolism than prokaryotically expressed pro21, possibly due to stabilization of the euk21 protein through Fc dimerization. We observed that the dulaglutide-FGF21 fusion had the highest activity, followed by the liraglutide-FGF21 fusion. Molecules fused with GIP, either the GIP-FGF21 dual agonist or the GLP-1 (dulaglutide or liraglutide)-GIP-FGF21 triple agonists, were basically ineffective under our experimental conditions.

Incretin receptor agonists such as tirzepatide offer effective pharmacological control of obesity but may be less effective for the treatment of NASH [7]. FGF21 analogs are effective for the treatment of NASH [ 4, 8]. Our study indicated that D21 has a significant impact on glucose and lipid metabolism, body weight and liver function. Incretin-FGF21 combinatory agonists may effectively control common metabolic diseases and thus warrant further investigation.

## Supporting information

23442Supplementary_Figures

## Supplementary Data

Supplementary data is available at *Acta Biochimica et Biophysica Sinica* online.
