# Supplementary material for: Incretin-FGF21 fusion molecule maximizes metabolic effects in mice: Incretin-FGF21 maximizes metabolic effects in mice
Source: Acta Biochim Biophys Sin (Shanghai). 2023 Dec 14;56(1):144–7. doi: 10.3724/abbs.2023276 (PMC10875359; doi:10.3724/abbs.2023276)
Supplement: 23442Supplementary_Figures [file 23442Supplementary_Figures.pdf]

A

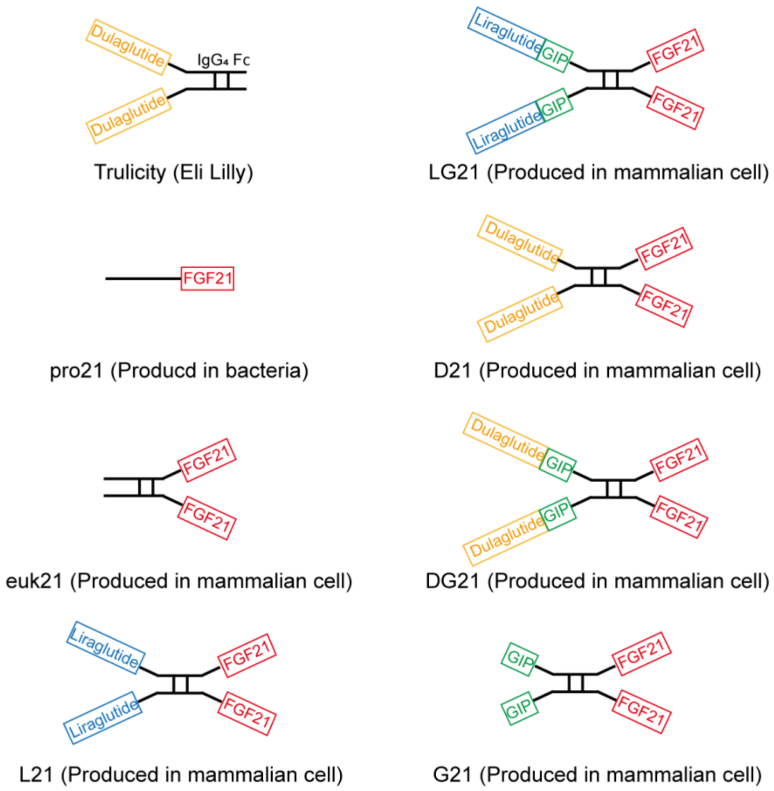

B

Dulaglutide
HGEGTFTSDVSSYLEEQAAKEFIAWLVKGGG

Liraglutide
HAEGTFTSDVSSYLEGQAAKEFIAWLVRGRG

GIP
YAEGTFISDYSIAMDKIHQQDFVNWLLAQKGKKNDWKHNITQ

FGF21 (DDKJ-102)
DSSPLLQFGGQVRQRYLYTDDAQQTEAHLEIREDGTVGGAADQSPESLLQLKALKPGVI
QILGVKTSRFLCQRPDGALYGSLHFDPEACSFRERLLEDGYNVYQSEAHGLPLHLPGNKS
PHRDPAPRGPARFLPLPGLPPALPEPPGILAPQPLETDSMDPFGLVTGLEAVRSPSFEK

Supplementary Figure S1. Structure and sequence of fusion proteins (A) Diagrammatic structure of Trulicity, pro21, euk21, L21, LG21, D21, DG21 and G21. (B) Sequence of the dulaglutide, liraglutide, GIP, and FGF21 analog (DDKJ-102).

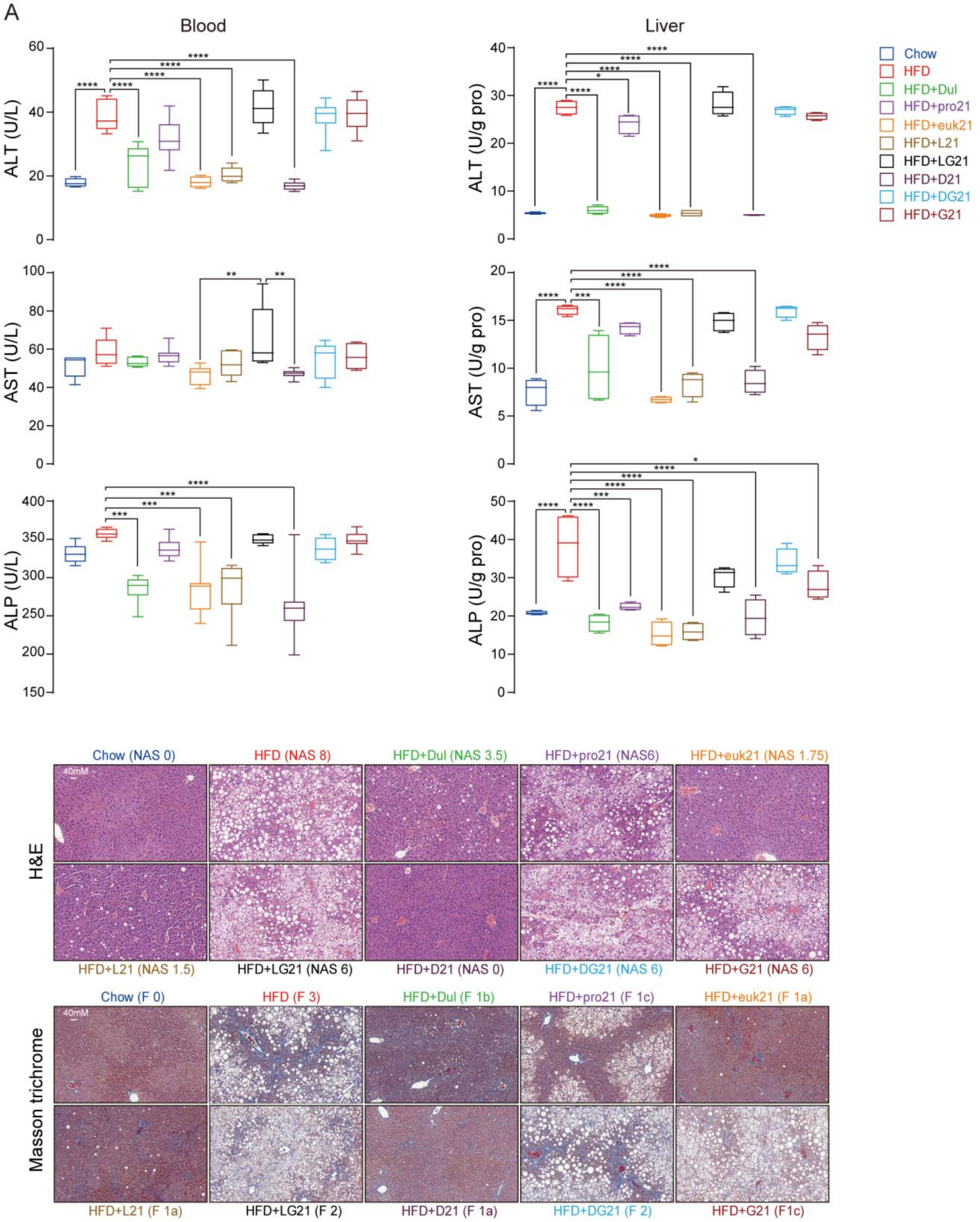

Supplementary Figure S2. Effects of fusion proteins on liver function (A) Box and whisker graph of blood and liver levels of alanine aminotransferase (ALT), aspartate aminotransferase (AST), and alkaline phosphatase (ALP). (B) Nonalcoholic fatty liver disease activity scores (number within parenthesis) and representative H&E histological images. Fibrosis scores (number within parenthesis)

and representative Masson trichrome staining images.
